# The Role of Ubiquitin and Ubiquitin-Like Modification Systems in Papillomavirus Biology

**DOI:** 10.3390/v6093584

**Published:** 2014-09-24

**Authors:** Van G. Wilson

**Affiliations:** Department of Microbial Pathogenesis and Immunology, College of Medicine, Texas A&M Health Science Center, 8447 HWY 47, Bryan, TX 77807, USA; E-Mail: wilson@medicine.tamhsc.edu; Tel.: +1-979-436-0310; Fax: +1-979-436-0086

**Keywords:** Ubiquitin, SUMO, human papillomaviru, proteasome, cervical cancer

## Abstract

Human papillomaviruses (HPVs) are small DNA viruses that are important etiological agents of a spectrum of human skin lesions from benign to malignant. Because of their limited genome coding capacity they express only a small number of proteins, only one of which has enzymatic activity. Additionally, the HPV productive life cycle is intimately tied to the epithelial differentiation program and they must replicate in what are normally non-replicative cells, thus, these viruses must reprogram the cellular environment to achieve viral reproduction. Because of these limitations and needs, the viral proteins have evolved to co-opt cellular processes primarily through protein-protein interactions with critical host proteins. The ubiquitin post-translational modification system and the related ubiquitin-like modifiers constitute a widespread cellular regulatory network that controls the levels and functions of thousands of proteins, making these systems an attractive target for viral manipulation. This review describes the interactions between HPVs and the ubiquitin family of modifiers, both to regulate the viral proteins themselves and to remodel the host cell to facilitate viral survival and reproduction.

## 1. Introduction –— Human Papillomaviruses

The human papillomaviruses (HPVs) are important pathogens with clinical manifestations ranging from benign to malignant lesions [1,2]. Over 170 different types have been sequenced with many more isolates identified but not yet classified [3]. The existing HPVs are classified into five genera, Alphapapillomaviruses, Betapapillomaviruses, Gammapapillomaviruses, Mupapillomaviruses, and Nupapillomaviruses, with the majority falling into genus alpha and genus beta. Most of the alpha group viruses infect mucosal epithelium while the beta types exhibit tropism primarily for cutaneous epithelium. Among the mucosal types a functional distinction exists between types that are highly associated with the development of cervical cancer, the so-called high-risk types, and the remaining nononcogenic, low-risk types. Because of their contribution to the development of cervical cancer, research has often focused heavily on the high risk types, particularly the most prevalent types, 16 and 18. More recently, a growing body of evidence linking cutaneous beta type HPVs to skin cancers [4] has led to increased examination of the biology and molecular biology of the beta HPVs. What has arisen from studies across multiple HPV types is that there are both common and type-specific features reflecting the great diversity of types and their biological niches [5,6,7].

**Figure 1:**
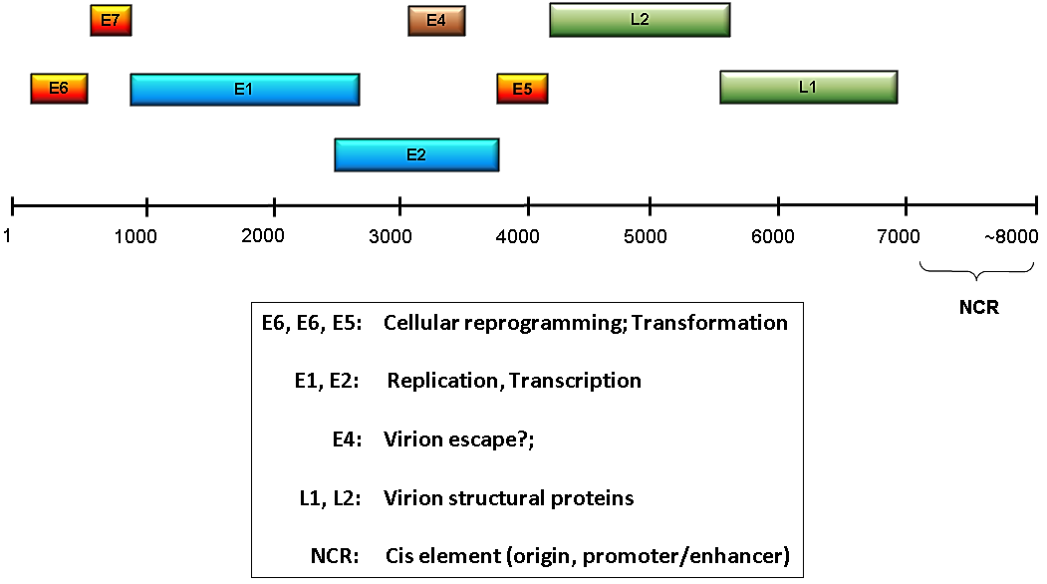
HPV genome organization and function. Shown is a linearized HPV genome with the relative genomic locations of the early (E) and late (L) ORFs indicated. The NCR is the noncoding region that contains the viral replication origin and transcriptional regulatory elements. Functions associated with the various ORFs are listed in the box below the genome.

A common feature of all papillomaviruses is a small, double-stranded, circular genome of about 8000 base pairs containing a single origin of replication [8]. These small genomes encode a limited number of proteins that are classified as either early or late depending on their time of expression. The early region of the genome contains open reading frames (ORFs) E1, E2, E4, E5, E6, and E7, and the early protein functions include viral genome replication, transcriptional regulation, and modulation of the host cell environment to favor viral replication and/or persistence (Figure 1). E6 and E7 gene products are particularly important for reprogramming the host cell, and for the high risk HPVs their E6 and E7 products constitute the predominant oncoproteins. There are only two late proteins, L1 and L2, and these are the viral capsid structural proteins [9,10]. Given the very limited repertoire of viral proteins, the HPVs have evolved to be highly dependent upon the host cells to supply needed functions and enzymatic activities. Much more detailed information about HPV proteins and their functions can be found in several recent reviews [11,12,13,14,15,16].

HPVs have a complex lifecycle that is intimately connected to the differentiation program of skin [17]. Establishment of infection requires viral entry into the replicative basal cells where the viral genome can persist episomally in the host cell nucleus [18]. Subsequent stages of the productive lifecycle occur in the subrabasal layers as differentiation progresses. Normally, these differentiating cells are nondividing and would not provide the replicative machinery and S phase environment required by the HPVs for their DNA replication, thus, a requirement of these viruses is to push the cells back into a proliferative state [19]. Additionally, HPVs must ensure appropriate expression of the viral proteins at different differentiation stages, effective assembly of new virions, and avoidance of host defense mechanisms [20]. Surprisingly, these viruses have only one enzymatically active protein, E1, which has helicase activity for viral genome replication [21,22,23]. Consequently, the actions of the other viral proteins are mediated entirely through protein-protein interaction, and this review will focus on how the HPV proteins interact with the ubiquitin and related ubiquitin-like modification systems.

## 2. Overview of the Ubiquitin Superfamily

**Figure 2:**
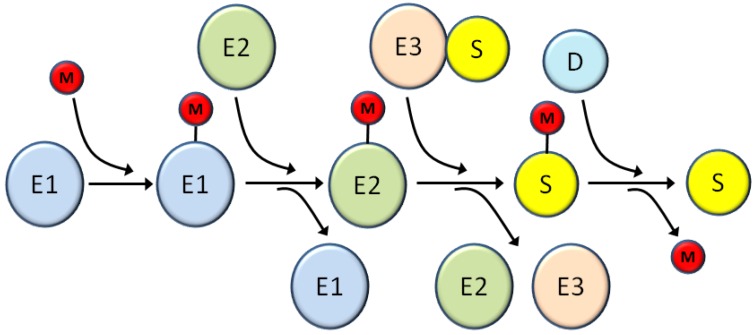
Schematic of the general cascade for modification by ubiquitin and ubiquitin-like (Ubl) modifiers. E1, E2, and E3 are the activating enzyme, conjugating enzyme, and ligase, respectively for the various Ubl pathways; the number of distinct enzymes at each step various for the particular modifier system. M represents ubiquitin or any other Ubl. S is the substrate and D is the demodifying enzyme that removes the modifier and returns the substrate to the unmodified form. See the text for more details about each step in the modification/demodification process.

All viruses have common missions that they must accomplish in order to successfully reproduce, including optimizing viral gene expression, promoting viral genome replication, and avoiding or abrogating host defense systems. At each of these steps viruses typically have evolved numerous complementary and/or redundant mechanisms to co-opt cellular proteins and machinery to support the viral lifecycle. Because of this dependence on host cells, viruses often target cellular systems with broad and important regulatory functions such as the ubiquitin super family [24]. This superfamily is characterized by small protein modifiers that are covalently attached to proteins substrates through a series of biochemically similar steps (Figure 2). These modifiers include ubiquitin and a series of ubiquitin-like proteins (Ubls), such as SUMOs [25], ISG15 [26], NEDD8 [27], FAT10 [28], and others [29]. The first step in the conjugation process is activation of the modifier through an ATP-dependent reaction that forms a thioester linkage between the modifier and the activation enzyme (E1) [30]. Subsequently the modifier is transferred to the conjugation enzyme (E2), again through a thioester linkage [31]. Finally, the modifier is transferred to the substrate using an E3 ligase that provides substrate specificity [32]. This final conjugation of the modifier to the substrate occurs through an isopeptide bond linking the C-terminus of the modifier with the epsilon amino group of a lysine residue in the target protein. Depending on the substrate, more than one lysine may be modified and more than one type of modifier may be utilized; in some cases the different modifications occur on separate lysine residues while in other cases different modifiers compete for the same lysine, often with opposing functional effects [33,34]. Additionally, depending on the modifier used both mono- and poly-chains of the modifier can be formed resulting in different outcomes for the substrate [35,36]. 

The prototype of this superfamily is ubiquitin, a 76 amino acids protein. Polyubiquitination targets proteins for proteosomal degradation and is a key cellular mechanism for removing damaged proteins and for regulating protein activity [37]. Ubiquitin chains can be removed by deubiquitinating enzymes (DUBs), thus, the balance between the modification and demodification reactions can be important for controlling substrate levels, including viral proteins [24,38]. In addition to ubiquitin, the SUMOs [39,40] and ISG15 [41] are the other two Ubls that have been implicated as modifiers involved in HPV viral processes. There are four human SUMO genes encoding SUMOs 1-4 [42], though SUMO 4 appears to have limited tissue expression [43] and has been much less studied than the other three. The processed forms of SUMOs 2 and 3 are 97% identical, while both are approximately 50% identical to SUMO1. While highly similar and often treated interchangeably in many studies, functional and biological differences have emerged for the three predominant SUMO types [44,45,46,47]. Consequently, the possibility of type-specific effects on substrates should be kept in mind when interpreting sumoylation studies. Like ubiquitin, SUMO moieties can be removed from substrates by a set of desumoylating enzymes known as SENPs (sentrin proteases) [48]. Lastly, mature ISG15 is a 157 amino acid ubiquitin-like modifier whose expression is highly stimulated by type I interferon [26]. While ISG15 modification is now known to impact a variety of cellular processes, its original discovery as an interferon-stimulated gene has focused a great deal of work in this field towards its role in viral infections where it has an antiviral effect [41].

## 3. Modification of HPV Viral Proteins by Ubiquitin and Ubiquitin-Like Modifiers (Ubls)

As for cellular gene products, the expression levels of viral proteins need to be highly regulated to coordinate their individual protein activities with the functional stages of the viral life cycle. This regulation typically occurs at both transcriptional and post-transcriptional levels. For HPVs, the viral early proteins tend to be expressed at relatively low levels and have short half-lives (Section 3.1., Section 3.2., Section 3.3., Section 3.4., Section 3.5. and Section 3.6.), while the L1 [49] and L2 [50] late proteins are much more stable (Section 3.7.). Proteasome inhibitor studies showed that the instability of these viral early proteins is proteasome dependent, and studies on individual HPV early proteins have shown that ubiquitination is a key element in targeting these proteins to the proteasome [51,52,53,54]. In combination, these results indicate that the ubiquitin-proteasome system is a key mechanism used by HPVs to regulate the intracellular levels of the viral gene products at the post-translational level. In addition to modification by ubiquitination, several HPV proteins are modified by sumoylation [39]. Sumoylation does not directly lead to degradation of the viral substrates, but does modulate biological functions. Both the ubiquitin and SUMO systems are utilized by the host cell to regulate aspects of cell cycle [55,56,57] and differentiation [57,58], and HPVs presumably take advantage of these systems to coordinate the levels of viral proteins with critical aspects of host cell cycle and epithelial cell differentiation. Alternatively, in some cases, these Ubl modifications may be antiviral in nature as the host cell attempts to reduce the level or activities of viral proteins as a protective measure. More specific details concerning the modification of the individual early proteins by ubiquitin and SUMO are presented below.

### 3.1. The E1 Proteins

E1 proteins are replication helicases that nucleate the formation of viral genome replication complexes and are essential for papillomavirus replication [13]. An early study revealed that the bovine papillomavirus (BPV) E1 protein was an unstable protein that was ubiquitinated and degraded via proteasomes [51]. In these studies, E1 was stabilized by association with the cyclin E/Cdk2 complex, apparently simply through complex formation, as Cdk2 kinase activity and E1 phosphorylation were not required for the protection from degradation. Under conditions which supported BPV origin-dependent DNA replication, E1 again became rapidly degraded, possibly as a consequence of dissociation from the cyclin E/Cdk2 complex. The elongation phase of DNA synthesis was required for E1 degradation, suggesting that E1 may dissociate from the cyclin E/Cdk2 complex at the completion of each round of genome replication. Subsequently, the anaphase-promoting complex (APC) was shown to be a primary E3 ligase for E1, specifically the APCCdh1 complex [59]. E1 interaction with, and degradation mediated by, the APC complex was dependent upon a KEN box and a D box within E1. As these motifs are highly conserved among HPV E1 proteins this suggests that the APC complex may be a universal regulator of E1 stability. However, comparable studies have not been directly done for HPV E1 proteins, thus, virtually nothing is known about the specific components of the ubiquitin-proteasome system that promote degradation of the human papillomavirus types.

In addition to modification by ubiquitin, E1 proteins are also substrates for SUMO, first shown for BPV E1 [60] and then for HPV 11 [61]. E1 sumoylation requires direct interaction with the SUMO conjugase, Ubc9 [60], and the interaction appears to be enhanced by E1 oligomerization [62]. Sumoylation of E1 is also stimulated by three SUMO ligases of the PIAS (protein inhibitor of activated STAT) family, suggesting that some or all of the members of this family may be the authentic *in vivo* ligases for E1 proteins [61]. While the available evidence strongly supports that E1 proteins can be sumoylated as a consequence of their interaction with Ubc9, the extent of E1 sumoylation is limited and the functional consequences of this modification are unclear. Sumoylation was originally reported to be required for nuclear localization of E1 [63], however, subsequent studies did not confirm this phenotype [62,64]. Intriguingly, all E1 mutants defective for Ubc9 binding are also impaired for DNA replication [62]. This could indicate that E1 sumoylation is required for some step in viral genome replication, but could also reflect requisite sumoylation of some other replication factor, such as PCNA (proliferating cell nuclear antigen) [65]. In this latter case E1 could be recruiting Ubc9 to the replication complex to redirect its activity to a host substrate critical for the viral replication process.

### 3.2. The E2 Proteins

E2 is a central regulatory factor for papillomaviruses and its expression is tightly regulated at multiple levels, including protein turn over. Ubiquitination and proteosomal degradation leading to a short half-life was first demonstrated for bovine papillomavirus E2 [66]. Similar ubiquitination and proteosomal degradation leading to short half-lives has been shown for E2 proteins from both high risk (types 16, 18, and 31) and low risk (types 6 and 11) HPVs, and their rapid degradation is dependent upon sequences within the amino terminal transactivation domain [52,67,68]. Importantly, E2 protein levels are cell cycle regulated with degradation occurring specifically at the end of G1 phase, and this degradation is mediated at least in part via interaction with the SCF^Skp2^ ubiquitin ligase [69]. High risk E2 proteins also associate with the Mdm2 ligase [70] and the APC/C ubiquitin ligase [71], though E2 does not appear to be a direct substrate for either ligase. For APC/C, E2 interacts with the activators, Cdc209 and Cdh1, and inhibits APC/C activity leading to the stabilization of several substrates involved in cell cycle control and chromosomal instability, including Skp2 [71]. This suggests a possible feedback mechanism to control E2 levels in cycling basal keratinocytes whereby E2 acts on APC/C to increase Skp2 which subsequently leads to E2 degradation via the SCF^Skp2^ ligase [69]. In contrast, in differentiated keratinocytes where Skp2 is not expressed this feedback would absent thus contributing to the observed increase in E2 levels in the upper layers of the epithelium [72].

While HPV 18E2 was shown to interact with the SCF^Skp2^ ubiquitin ligase that contains cullin1, HPV 16E2 associated with cullin3 but only weakly if at all with cullin1 [73]. Inhibition of cullin3-based E3 ligases with a dominant-negative CUL3 led to reduced ubiquitination and a significantly increased half-life for 16E2, suggesting that 16E2 degradation is mediated via a cullin-3 containing ubiquitin ligase. Whether or not 18E2 and 16E2 are actually ubiquitinated via different E3 ligases, or if there is redundancy in which ligases can target E2 proteins, remains to be determined. Interestingly, Brd4, an activator of E2 transcriptional activity, blocks the interaction between E2 and cullin-3 resulting in increased stability of E2, presumably through reduced ubiquitination [73]. Similar stabilization by Brd4 has been reported for the E2 proteins from bovine papillomavirus [74], HPV 11 [68,75], and HPV 31 [68]. Brd4 directly binds to transactivation domain (TAD) of E2 which suggests that Brd4 may be a universal regulator of E2 stability through competition with E3 ligase complexes that ubiquitinate the TAD domain [68]. Alternatively, a recent report suggests that E2 is primarily ubiquitinated in the cytoplasm and that Brd4 stabilizes E2 by sequestering it in the nucleus, where it is not accessible for degradation [76].

In addition to Brd4, several other proteins have been shown to increase E2 half-life by preventing proteosomal degradation, including two cellular proteins, Tax1BP1 [77] and NRIP [78], and two HPV proteins, E1 [79] and E1^E4 [80]. None of these proteins had any significant effect on E2 transcript levels, thus, they appear to be acting at the protein level. For the two viral proteins, E1 and E1^E4, no mechanism was explored. Tax1BP1 is a subunit of an ubiquitin-editing enzyme complex [81] and binds E2 through the TAD region, consistent with Tax1BP1 potentially acting to protect E2 by deubiquitinating this domain. However, no reduction of E2 poly-ubiquitination was observed in the presence of Tax1BP1, so the mechanism of E2 stabilization remains undefined. Like Tax1BP1 and Brd4, NRIP also interacts with E2 through the TAD domain [78]. Unlike Tax1BP1, NRIP was able to reduce ubiquitination of 16E2 and this was related to dephosphorylation of E2 mediated via recruitment of calcineurin by NRIP. Exactly how phosphorylation promotes or contributes to ubiquitination remains unclear, especially as other reports suggest that phosphorylation may stabilize 16E2 [82] and 8E2 [83]. An intriguing suggestion is that phosphorylation may regulate chromatin association leading to bound E2 that is protected from degradation, possibly by sequestration from the ubiquitination machinery, and is consequently much more stable than unbound E2 [83]. In this model phosphorylation could be a determinant of either stability or degradation depending on how it affected chromatin binding by a particular E2 type and during a particular cell cycle stage.

Lastly, there is evidence that E2 degradation is subject to crosstalk between the ubiquitin system and the sumoylation system [84]. BPV, HPV 11, HPV 16, and HPV 18E2 proteins have all been shown to bind Ubc9, the SUMO conjugating enzyme, and the 16 and 18E2 proteins could be sumoylated *in vitro* and *in vivo* [85]. Type 16E2 was tested for functional effects, and sumoylation was found to contribute to both transactivation and repressive activity. In addition to these functional effects, increasing overall intracellular sumoylation levels led to a dramatic stabilization of 16 and 18E2 proteins via prevention of proteosomal degradation [86]. Surprisingly, the stabilization mediated by increasing overall sumoylation did not occur through direct sumoylation of the E2 proteins themselves as sumoylation defective E2 mutants were stabilized as effectively as the wild-type protein. This indirect effect on E2 degradation suggests that increasing sumoylation regulates some component of the ubiquitination/deubiquitination pathway to prevent proteosomal degradation. Biologically this may contribute to enhanced E2 expression in suprabasal layers of the skin where sumoylation is increased [87]. In summary, the overall levels of E2 reflect a complex interplay between numerous factors that can interact with E2 to facilitate or inhibit degradation, and there appear to be type specific nuances that further confound our current understanding.

### 3.3. The E4 Proteins

The E4 ORF is located within the E2 gene and is expressed primarily as an E1^E4 product [16]. The E1^E4 protein is expressed at relatively high levels during productive viral replication and accumulates in the mid to upper epithelial layers, suggesting that it is a fairly stable protein [88,89]. While modification of E1^E4 by kinases [90,91,92] and proteases [93,94] is known, modification by the ubiquitin superfamily members has not been reported.

### 3.4. The E5 Proteins

The E5 protein is the major oncoprotein for BPV and a contributor to the oncogenic process in HPVs [14]. It is a very small (44 amino acids for BPV and approximately 80 amino acids for HPVs), hydrophobic, and membrane associated protein that is expressed at very low levels. Like E4 proteins, the turnover and degradative mechanisms have not been explored in any detail, thus, whether or not it is modified by ubiquitin for proteosomal targeting is unknown.

### 3.5. The E6 Proteins

E6 proteins from high-risk HPV types are moderately short-lived proteins [95,96] that are degraded via the proteasome [53,97]. The degradative mechanism for other E6 proteins is less certain as an initial study reported that two low-risk genital type E6 proteins (6a and 11) were not stabilized by a proteasome inhibitor and two cutaneous HPV E6 types (5 and 8) showed only slight stabilization by the inhibitor [53]. However, a subsequent study found that type 11E6 was ubiquitinated and degraded via the proteasome, suggesting that all E6 types may share a common degradative pathway [97]. Differences in epitope tagging or cell type for the expression experiments may account for these conflicting results, and further studies are needed to clarify possible differences in degradative mechanisms between various E6 types. In contrast, both studies agreed that this degradative process does not require interaction with E6AP, a known ubiquitin E3 ligase that binds E6 proteins [98]. Interestingly, a latter study found that the presence E6AP actually stabilizes both HPV 16 and 18E6, though the E6AP enzymatic activity is unnecessary for this protective effect [99], and similar results were later found for cutaneous HPV E6 proteins [100]. The combined results infer that E6AP is not the E3 ligase responsible for ubiquitinating E6 itself, that E6AP is acting through some other mechanism to affect E6 turnover, and that this mechanism may be common to all E6 types. The true E3 ligase(s) for E6 proteins remains undetermined, but other possible candidates have been identified through recent proteomic approaches. HERC2, a putative E3 ligase of the HECT-domain type, was associated with HPV 16E6 [101], a HECT-domain E3 ligase, called EDD, binds strongly to HPV 18E6 and weakly to type 16 and 11 E6 proteins [102], and some E6 proteins from genus beta interact with the Ccr4-Not complex which has known ubiquitin ligase activity [6]. However, none of these candidates has been functionally tested for ability to ubiquitinate E6, thus, all may simply be E3 ligases recruited to E6 to facilitate modification of other substrates rather than E6 itself. For example, EDD enhances ubiquitination of E6AP and reduces E6AP levels which indirectly reduces E6 levels, but does not appear to affect E6 directly [102]. Likewise, HERC2 is known to stimulate the ligase activity of E6AP [103], so could be acting to enhance the activity of the E6-E6AP complex rather than targeting E6 itself. No such HERC2 enhancing effect was observed for E6-mediated p53 degradation in HeLa cells [104], but this does not rule out a possible HERC2 effect on other E6 targeted substrates.

Lastly, in addition to binding E3 ligases, HPV 16E6 binds a deubiquitinating enzyme, USP15, and both 16 and18E6 proteins are stabilized by catalytically active, but not an inactive, mutant of USP15 [101]. These results suggest that USP15 is protecting E6 proteins by removing ubiquitin chains and are consistent with a typical ubiquitin-proteasome pathway for degradation of at least the high-risk HPV E6 proteins. Somewhat surprisingly, HPV 16E6 is also stabilized by association with PDZ domain proteins [105]. As PDZ proteins are themselves targets for E6-E6AP mediated degradation [106,107], this observation suggests that the complex network of interactions between E6 and its associated proteins can influence E6 stability and proteolysis, similar to what has been observed for E2 proteins.

### 3.6. The E7 Proteins

Like its partner oncoprotein, E6, the HPV E7 proteins are also short-lived with HPV 18E7, initially shown to have a half-life of less than 15 minutes in HeLa cells [108]. Subsequent studies of HPV 16E7 in Cos 7 cells [54] and CaSki cells [109] both showed that E7 was ubiquitinated and that its short half-life was due to proteosomal-dependent degradation. The 16E7 half-life in Cos7 cells was 30-40 minutes, consistent with the high-risk E7 proteins, generally being short-lived proteins. Interestingly, mutational interrogation revealed that E7 degradation required the N-terminal eleven amino acids of E7 which directed N-terminal poly-ubiquitination of E7; neither of the two internal lysines in E7 was involved [54]. Tandem mass spectrometry (MS/MS) analysis of ubiquitinated HPV58 E7 confirmed N-terminal conjugation of the ubiquitin moiety suggesting that this may be a general mechanism for E7 ubiquitination [110].

While ubiquitin-mediated, proteosomal degradation of E7 proteins is well established, less certain are the components of the ubiquitination machinery that are specifically involved in E7 modification as several candidates have been identified. The first potential candidate identified was SOCS1 which binds E7 *in vitro*, co-localizes with E7 *in vivo*, stimulates E7 ubiquitination, and promotes proteosomal degradation of E7 [111]. SOCS1 is a component of the VCB-type ubiquitin E3 ligase that appears to function as an adapter protein to target this ligase to substrates [112], implying that SOCS1 is part of an E3 ligase complex that controls E7 degradation. However, it was noted the E7 was still partially ubiquitinated in the presence of an inactive SOCS-box defective mutant of SOCS1, suggesting that additional E3 ligases could be involved [111]. Consistent with this observation, Oh *et al.* demonstrated that the Cull1-Skp2 E3 ligase could also bind E7, function to ubiquitinate E7, and mediate E7 proteosomal degradation [113]. Like the SOCS1 study, E7 degradation was only partially blocked in the absence of Skp2, thus, at least two E3 ligase pathways for E7 proteolysis seem plausible. Additionally, Oh *et al*. identified UbcH7 as a functional E2 ligase for E7 while seven other E2 ligases (UbcH2, UbcH3, Ubc5A, Ubc5B, Ubc5C, UbcH6, and UbcH10) were ineffective. Unfortunately, later studies have not demonstrated an association of E7 with cullin 1 [5,114], thus, the authentic E3 ligase complexes that ubiquitinate E7 remains uncertain.

In addition to the dual E3 ligases that have been reported to facilitate E7 degradation, there is one report of a deubiquitinating enzyme (DUB) specific for E7, USP11 [115]. Both *in vitro* and *in vivo*, USP11 binds E7 and reduces the extent of poly-ubiquitination. Over expression of USP11 increases the half-life of E7 while siRNA knockdown of USP11 enhanced E7 degradation with a concomitant loss of E7 transforming activity in CaSki cells. While these studies do not rule out the existence of other DUBs that could contribute to the overall balance of ubiquitinated *versus* unmodified E7, they do strongly implicate a role for USP11 in at least some cell types.

### 3.7. L1 and L2 Proteins

The L1 and L2 capsid proteins are relatively stable and there are no reports of ubiquitination for either protein [9,10]. However, HPV16 L2 can be sumoylated at lysine 35 with a strong preference for SUMO2/3 over SUMO1 [50,116], and HPV16 L1 can be modified with ISG15 [117]. ISG15 modified L1 was incorporated less effectively into pseudovirion particles and the resulting modified pseudovirions had significantly reduced infectively, demonstrating that this modification could be regulating virion production during an authentic infection. Similarly, sumoylation of L2 prevents binding to L1, thus, sumoylation may also contribute to regulation of capsid assembly [50]. Sumoylation of L2 did not prevent binding to E2, but the effects of this modification on other known interactions of L2 were not examined. Interestingly, a lysine 35 to arginine mutant L2 protein had reduced stability compared to the wild type protein. However, the study did not distinguish whether sumoylation at lysine 35 actually protects L2 from degradation or that the arginine at position 35 simply makes L2 intrinsically less stable. Additionally, neither sumoylation nor ISGylation has been examined in the context of normal keratinocytes, so the presence and potential function of these modifications during the normal viral life cycle has not been verified.

## 4. Modulation of the Host Environment Mediated Through HPV-Ubl Interactions 

In addition to utilizing the ubiquitin-proteasome system, along with other Ubls, to regulate turnover of HPV polypeptides themselves, several HPV proteins have targeted these systems to control the levels and activities of critical cellular proteins. The ubiquitin superfamily, particularly ubiquitin and SUMO, modify hundreds to thousands of host proteins [118,119]. Redirecting these ubiquitous post‑translational modifications systems provides the virus with enormous opportunities for reprogramming the cellular environment to favor viral persistence and reproduction. For HPVs, none of the viral proteins has intrinsic E3 ligase or deubiquitinating activity, so manipulation of these Ubl systems requires interaction between HPV proteins and cellular components of the ubiquitin or SUMO pathways. Retargeting host E3 ubiquitin ligases to new substrates to facilitate degradation of selected host proteins is an important mechanism for some HPV early proteins, particularly E6 and E7 [120]. Alternatively, some early proteins interfere with E3 ligases or recruit DUBs to prevent degradation of host proteins. Examples of how HPV proteins enhance, inhibit, and redirect the Ubl systems are presented below.

### 4.1. The E1 Proteins

While little is known about the specific enzymes involved in HPV E1 ubiquitination and proteasomal degradation, a recent association with a deubiquitinating complex has been described [121,122,123]. E1 proteins from anogenital papilloma virus types, but not cutaneous types, interact with a host protein originally named p80 and, subsequently, called UAF1 [121]. UAF1 is a cytoplasmic protein that is recruited to the viral origin in an E1- and E2-dependent fashion and is necessary for wild-type levels of viral DNA replication [122]. However, UAF1 is not required for assembly of the E1-E2-ori complex so must be acting at a downstream point in the replication process. In addition to binding viral E1, UAF1 interacts with three deubiquitinating enzymes, USP1, USP12, and USP46, leading to formation of a ternary complex on the viral origin, consisting of E1, UAF1, and any one of the three DUBs [123]. The presence of a DUB in the complex is as critical for replication activity as is UAF1 itself, suggesting that the primary role of UAF1 is to recruit a DUB to the replication initiation complex. What is missing at this point is the mechanism by which a DUB facilitates replication and the relevant target(s) for the DUB. Removal of ubiquitin from E1 to increase E1 levels is one possible mechanism to enhance replication, but the UAF1-USP complex is known to target other substrates involved in DNA replication and chromatin structure [124,125,126], thus, experimental determination of the functional targets will be needed to fully resolve how E1 utilizes host DUBs to promote viral genome replication. If the target(s) is not E1 itself, this would be a new example of how certain HPVs have usurped the ubiquitin system to target host proteins in ways that facilitate the viral life cycle.

### 4.2. The E2 Proteins

E2 is the major transcriptional regulatory protein for papillomaviruses, and it can exert either positive and negative effects on viral promoters depending on the context of E2 binding sites [12]. Over 80 host cell proteins have been identified as partners of various E2 types, many of them involved in transcriptional regulation and chromatin modification/remodeling as expected for a viral transcriptional regulatory protein. Among this group of known E2 partners is the E3 ligase, Mdm2 [70]. Mdm2 binds to C-terminal DNA binding domain of E2, can complex with E2 on E2 binding sites, and activates E2-dependent transcription of the HPV 16 promoter. While transcriptional activation requires the E3 ligase activity of Mdm2, E2 protein levels are unaffected by Mdm2 suggesting that E2 is not a direct substrate for Mdm2. Thus the transcriptional effects appears to be mediated via another mechanism involving ubiquitination, though the specific target for the E2-Mdm2 complex is still unknown. Potentially Mdm2 could be targeting other proteins associated with the HPV 16 promoter for proteolysis or affecting protein-protein interactions at the promoter, as is well established for other transcriptional units [127].

Perhaps because of a focus on E2’s transcriptional function, there has been very limited investigation of E2 interactions with the ubiquitin-proteasome system outside the context of transcriptional control. As mentioned in Section 3.2., high-risk E2 proteins type 16 and 18, but not low-risk E2s, associate with Cdc20 and Cdh1 (also known as FZR1) which are activators of the Anaphase Promoting Complex (APC), an important E3 ligase regulating cell cycle [71]. E2 appears to sequester Cdh1 into insoluble cytoplasmic structures, thus preventing APC activation and allowing APC substrates, such as cyclin B, to escape degradation. This dysregulation of APC ultimately leads to genomic instability which may contribute to the oncogenic potential of high-risk HPVs.

The two above example confirm that E2 proteins can in some instances function as modulators of the ubiquitin-proteasome system, but until recently this mechanism seemed to be relatively limited for E2 proteins. However, more global and unbiased evaluations of large-scale protein-protein interaction networks for multiple E2 protein types have revealed a more widespread connection between E2 and the ubiquitin-proteasome system [128,129]. A yeast two-hybrid screen of 12 different E2 types from three clades against a human keratinocyte (HaCaT) cDNA library revealed that proteins in the ubiquitin functional family comprised a major target for E2 proteins with 26 partner proteins identified [129]. Among these partners were two members of the HECT domain family of E3 ligases (HUWE1, WWP2) and several adaptor proteins of cullin-based ubiquitin ligases (BTBD1, SPOP, CDC20, CDH1, FBX022). While some of these interactions may be involved in ubiquitination and turnover of E2 itself, it is likely that some reflect novel pathways, whereby E2 redirects the ligases to new targets or modulates the ligase activity on their normal targets. Additionally, the interactor list contained PIAS1 and PIAS4, E3 ligases in the sumoylation pathway [130]. As E2 is known to be sumoylated [85], these PIAS proteins could be acting as SUMO ligases for E2, or again E2 could be retargeting them to enhance sumoylation of E2 partners. Regardless of the outcome, these more global interaction studies suggest that the ability of E2 to manipulate members of the ubiquitin super family may be broader and more extensive than previously considered.

### 4.3. The E4 Proteins

E4 proteins are known to mediate keratin network disruption which is believed to comprise epithelial integrity and promote virion release [131]. There is one report that at least part of the mechanism for this E4-mediated disruption of the keratin network is through hyper-ubiquitination of keratin proteins that are associated with E1^E4 protein, which could lead to enhanced proteosomal degradation of the keratins [132]. This hyper-ubiquitination is likely to be an indirect effect triggered by E1^E4 induced phosphorylation of the keratins rather than direct recruitment of an ubiquitin E3 ligase by E1^E4.

### 4.4. The E5 Proteins

The HPV E5 oncoproteins are membrane bound and mediate their biological effects through association with several host cell proteins, including EGFR [133]. E5 enhances EGFR activity through multiple mechanisms, at least one of which appears to be interference with ubiquitin-proteasome degradation [134]. In this mechanism, HPV16 E5 reduced association of an E3 ligase, c-Cbl, with EGFR leading to a reduction in EGFR ubiquitination. The 16E5 effect on EGFR levels was similar to that seen by proteosomal inhibition, suggesting that interfering with the EGFR/c-Cbl interaction was an important contributor to E5 activation of EGFR. Similarly, 16E5 is reported to block the ubiquitin degradative pathway for keratinocyte growth factor receptor (KGFR), also by affecting the c-Cbl E3 ligase [135]. In this case E5 impairs phosphorylation at tyrosine 196 of the FRS2α docking protein that is constitutively associated with KGFR. Phosphorylation of this tyrosine is necessary to recruit the Grb2-Cbl ligase complex, so reduction of modification at this residue by E5 likely leads to reduced ability of the ligase to ubiquitinate the KGFR-KFS2 complex.

In contrast to the above studies showing an E5-mediated inhibition of proteosomal degradation for two growth factor receptors, there are now two reports that E5 proteins can also enhance degradation of certain host proteins. CD1d is an important major histocompatibility complex (MHC) type 1 glycoprotein that is down regulated at the protein level in HPV 6 or 16 E5 expressing cells; down regulation is abrogated by a proteasome inhibitor indicating a likely ubiquitin-proteasome degradative process [136]. In this case, E5 interacts with calnexin, an ER chaperone for CD1d, which likely results in misfolding of CD1d leading to its degradation. Decreased CD1d mediated by E5 results in abrogation of IL-12, a major cytokine important in both innate and adaptive immunity to pathogens, which would like aid in HPV evasion of the host immune response. While not a direct interaction between E5 and components of the ubiquitin-proteasome system, this example illustrates more subtle mechanisms by which the HPV proteins can influence the ubiquitin-proteasome system to the virus’ advantage. Another example of E5-mediated degradation has been reported for the pro-apoptotic protein, Bax [137]. Expression of HPV16 E5 increased ubiquitination of Bax leading to a decreased half-like that was rescued by a proteosomal inhibitor. E5 appeared to be acting through a pathway involving XOX-2, prostaglandin E2, and PKA, thus, again this is likely due to an indirect mechanism rather than a direct E5 interaction with components of the ubiquitin-proteasome system. As there are additional reports in the literature that E5 proteins can decrease the levels and/or half-lives of other cellular proteins [138,139], direct or indirect utilization of the ubiquitin-proteasome system by E5 may be a general feature of this viral protein.

### 4.5. The E6 Proteins

Historically, high risk E6 proteins are the prototype for HPV modulation of the host cell through the ubiquitin-proteasome system. Via their ability to bind the host ubiquitin ligase, E6AP, high risk E6 proteins retarget the ligase to modify E6 partners and promote the proteosomal degradation of these partners [11]. At least for the high risk E6 proteins, knocking down E6AP levels has nearly the same effect on global gene expression as knocking down E6 itself [140], indicating that a large portion of E6’s functional effects are mediated through E6AP, either utilizing its E3 ligase activity or a possible transcriptional activity [141]. Furthermore, E6AP is necessary for E6 to cause cervical cancer in a mouse model which strongly confirms that the oncogenic activity of the high risk E6 proteins is mediated through E6AP targets [142]. The initially described target of the E6-E6AP complex was the p53 tumor suppressor protein [143,144], and the ability of the high risk E6 proteins to promote p53 degradation remains a key element in the transforming activity of these proteins [145]. In addition to targeting p53, the high risk E6 proteins have a carboxy-terminal motif that can interact with cellular PDZ proteins and target their proteosomal degradation via E6AP [106,107,146,147,148]. The E6-targeted PDZ proteins that have been identified to date are membrane associated proteins involved in cell-to-cell adhesion and communication [149], thus, their dysregulation by E6-E6AP is likely to have significant effects on the host cells and may contribute to the oncogenic potential of the high-risk HPVs. However, the true extent to which these PDZ proteins are degraded *in vivo* during a natural infection and the actual biological consequences of such degradation remain largely unknown.

While most early studies on HPV E6 proteins focused on the high-risk types, due to their importance in human cancers, more recent studies have shown interesting similarities and differences among various E6 types. For example, interaction with E6AP has also been shown for low risk mucosal HPVs [7,150,151], and this conserved ability to interact with E6AP occurs through the LXXLL motif on E6AP as for high risk E6 proteins [152,153,154]. However, in contrast to high risk E6 proteins, the low risk mucosal types fail to interact with or significantly degrade p53 even though they can bind E6AP [144,151,155]. This observation at least partially explains their low oncogenic potential and suggests that there must be subtle differences in the resulting E6-E6AP complexes between low and high risk mucosal E6 proteins that can affect target discrimination. Similarly, the low risk E6 proteins lack the PDZ binding motif so these proteins are not degraded by low risk E6s. Nonetheless, since the E6-E6AP interaction is conserved between high and low risk E6 types, there are likely to be other E6-E6AP targets that are biologically important for all mucosal HPVs. 

In support of a broader role for the E6-E6AP complex in the normal life cycle of HPVs, many other E6 binding partners have been identified both through targeted studies of individual proteins and more recently through proteomics approaches [6,7,102,156,157], and many of these partners are proteasomally degraded in an E6AP-dependent fashion. Discussion of all the numerous identified E6-E6AP targets is beyond the scope of this review, but more comprehensive discussions of individual targets can be found in several recent reviews [11,158,159,160,161]. Furthermore, the general importance of this E6-E6AP interaction has been extended by the recent demonstration that E6 proteins from cutaneous HPVs of the beta genus associate with E6AP [162]. However, there is considerable variation between individual E6 types and the extent of association with E6AP [100] and at least one report that several beta type E6 proteins do not bind E6AP [6], thus, the true generality of the E6-E6AP complex remains unresolved. Consistent with a possible general role for E6-E6AP, there is one common E6-E6AP targets for nearly all tested E6 proteins, Bak [163,164,165]. Bak is a pro-apoptotic protein [166], thus, down regulating its activity through degradation may be a critical requirement for all HPVs. In contrast, the more type-specific targets for E6-E6AP may reflect unique requirements for viral persistence and/or reproduction in different types of epithelium. 

The well-established and widespread ability of E6 proteins to interact with and utilize E6AP as an E3 ligase to ubiquitinate host proteins for targeted degradation does not exclude interactions between E6 and other components of the ubiquitin-proteasome system. As described in Section 3.5., proteomics approaches have demonstrated associations of various E6 proteins with other E3 ligases, including HERC2 [101], EDD [102], and the Ccr4-Not complex [6]. What role these ligases play with E6 in the HPV life cycle, and what their potential targets might be, remain undefined. Additionally, there is accumulating evidence that E6 proteins can interact directly with proteosomal components. Two recent proteomics studies detected association of E6 proteins with multiple proteosomal subunits [6,7], and direct binding to multiple subunit proteins, including S2, S4, S6a and b, S7, S8, and S10 was demonstrated *in vitro* for 16E6, 11E6, and 18E6 [167]. E6 was also able to increase the ubiquitination of the S5a subunit through E6AP which increased S5a degradation [167]. How these proteosomal interactions may affect overall proteosomal function and/or degradation of E6 targets has not been examined, but does suggest that the utilization of the ubiquitin-proteasome system by E6 proteins is multi-faceted and complex.

One other intriguing aspect of post-translational regulation by E6 is cross-talk between the ubiquitin and SUMO systems. The initial observation was that 16E6 binds PIASy, a SUMO E3 ligase, and inhibits its activity [168]. This reduction of PIASy activity results in decreased sumoylation of specific PIASy substrates, including p53 and pRb. PIASy normally activates p53 and represses pRb, thus, the E6 mediated inhibition of PIASy would contribute to the reduced p53 activity and increased pRb activity in high risk HPV infected cells. Subsequently, a more global effect of E6 on the sumoylation system was demonstrated with the finding that the SUMO conjugating enzyme, Ubc9, is a substrate for E6AP-dependent degradation by high risk E6 proteins, leading to numerous changes in the cellular sumoylation profile [169]. Sumoylation is important in normal keratinocyte differentiation [87,170], thus, the ability of E6 to disrupt the sumoylation program broadly may contribute to the aberrant differentiation phenotype of high risk HPVs. Other more substrate specific possibilities exist, such as described in a recent study on hADA3, a transcriptional coactivator [171]. In the absence of E6, hADA3 is a normal substrate for E6AP, and the ubiquitination and subsequent degradation of hADA3 is enhanced by its increased sumoylation. Expression of high risk E6 further enhances sumoylation of hADA3 thus leading to its decreased stability. While enhanced sumoylation of hADA3 might seem to conflict with generally reduced sumoylation caused by E6 targeting of Ubc9, on an individual substrate basis there may be no conflict. For example, recruitment of E6 to the E6AP-hADA3 complex could retarget E6AP to cause degradation of an associated SUMO protease resulting in a net increase in hADA3 sumoylation. While speculative, such models illustrate the additional levels of complexity that arise through cross-talk between the Ubl systems. Significant further studies will be needed to untangle and understand the biological consequences of these processes. 

### 4.6. The E7 Proteins

Like E6, the characterization of E7 proteins has focused heavily on the major oncogenic types, 16, 18, and 31, with many fewer studies on the low risk alpha types or the beta types [15]. The primary target for the high risk E7 proteins that is associated with their oncogenic potential is the host pRb protein which is degraded through the ubiquitin-proteasome pathway in an E7-dependent fashion [172,173,174,175]. MDM2, the natural E3 ligase for pRB is not involved in the E7-mediated degradation of pRB [176]. Instead, for HPV16 E7, a cullin 2 ubiquitin ligase associates with the E6-pRb complex, leading to pRB ubiquitination and degradation [114]. 16E6 preferentially associates with the activated NEDD8 modified form of cullin 2, and may induce neddylation and/or stabilize the neddylated form of cullin 2 [114]. However, 16E6 does not appear to bind directly to cullin 2, and association with cullin 2 requires the substrate specificity factor, ZER1 [5]. Intriguingly, ZER1 interacts only with 16E7 and not 18E7, and 18E7 does not directly bind to cullin 2 [114], thus, apparently, 18E7 utilizes a different E3 ligase to target pRb for degradation. Consistent with a requirement for another E7-associated E3 ligase, proteomics studies identify several other E3 ligase components, including KCMF1, UBR4, and cullin 3, that associate more generally with E7 proteins from alpha and beta HPV types [5]. What role these other potential E3 ligase complexes play in E7 modulation of the host environment is currently unknown, but the conservation of these interactions suggests that there are common functions across most, if not all, HPV E7 types that require the ubiquitin-proteasome system. One example of such a target is the pRB family member, p130, which is an important regulator of differentiation, cell growth, and senescence [177]. P130 is proteasomally degraded in an E7-dependent fashion by both high and low risk alpha HPVs [178,179,180]. While the specific E3 ligase components involved are undetermined, this critical regulatory protein could be a mandatory target for mucosal E7 proteins to facilitate a replicative environment in terminally differentiating keratinocytes; there is very little information about the role of p130 for the cutaneous types, though HPV1 has been reported to destabilize p130 in raft cultures [181].

In addition to the direct targeting of host proteins through complex formation between E7, E3 ligases, and the substrate, E7 also indirectly influences the ubiquitin-proteasome system to modulate levels of some host proteins. Spardy *et al.* demonstrated that 16E7 accelerates claspin degradation which helps abrogate DNA damage checkpoint responses and facilitates mitotic entry [182]. Claspin is normally targeted for proteosomal degradation through ubiquitination by the SCF^β-TrCP^ type E3 ligase, and 16E7 up regulates several components of this complex presumably leading to increased ligase activity and enhanced modification/proteolysis of claspin. As the unregulated components all possess E2F-responsive promoter elements, it is likely that E2F activation resulting from E7-mediated degradation of pRb is the responsible for the increased expression of these components rather than a direct association of E7 with the SCF complex. A similar E2F-dependent mechanism appears to be involved in E7 regulation of the APC/C E3 ligase complex [183]. HPV16 E7 expression inhibits the degradation of APC/C substrates, such as cyclin A and cyclin B, during mitotic progression [184], and this inhibition is related to increased expression of EMI1, a negative regulator of APC/C [183]. At least part of the increased EMI1 levels results from increased transcription, again likely due to E2F activation following pRB degradation. Additionally though, EMI1 is also stabilized at the protein level by E7 though the precise mechanism has not been determined. This combination of transcriptional and post-transcriptional effects raises EMI1 levels sufficiently to abrogate normal APC/C function. Decreased APC/C activity has been proposed to be a general requirement to create a nuclear environment permission for viral replication [185], thus, targeting APC/C via E7 is likely to be functionally significant for high-risk HPVs. Together, these two examples clearly illustrate the E7 proteins can also manipulate the ubiquitin-proteasome system via indirect pathways that do not require direct interaction of E7 with the ubiquitination machinery. 

Lastly, like E6, high risk 16E7 can associate with at least one proteosomal component, S4 [186]. This same subunit is also up regulated by E7 [187], though no information is available about mechanisms or the functional significance of E7-S4 interaction. Nonetheless, this ability to interact with a proteosomal component suggests additional complexity in the relationship between E7 and the ubiquitin-proteasome system that needs further exploration. 

### 4.7. L1 and L2 Proteins

Modulation of the ubiquitin superfamily by L1 and L2 has not been reported, but two recent publications have identified intriguing interactions of L2 with the sumoylation system. Marušič *et al.* found the HPV16 L2 expression in U2OS cells caused an increase in nuclear SUMO2/3 accumulation that did not require sumoylation of L2 itself [50]. While the functional significance of this L2-mediated up regulation is unknown, sumoylation has been shown to increase and be necessary during normal keratinocyte differentiation [87] and to be decreased by high risk E6 proteins [169], so perhaps L2 is acting to modulate the E6 effect in the higher layers of the epithelium. In addition to modulating host sumoylation, HPV16 L2 has been shown to have a functional SUMO interaction motif (SIM) that directly binds SUMO2 more strongly than SUMO1 [116]. The functional SIM was localized to amino acids 284-289, and this sequence is highly conserved among the high-risk L2 proteins. Mutation of this SIM motif had no effect on capsid assembly, binding to host cells, or intracellular trafficking, but abrogated the normal association of the L2-DNA complex with PML bodies and greatly reduced infectivity of HPV 16 pseudoviruses. These results strongly suggest that the L2 protein has evolved a SIM motif to facilitate localization of the viral genome to PML bodies during in the initial infection process.

## 5. Conclusions

Human papillomaviruses have evolved an incredibly varied and complex set of interactions with the host cell ubiquitin-proteasome system and some of the related Ubl modifier systems. Since the discovery of the E6-E6AP complex and its role in p53 ubiquitination and degradation 20 years ago, there is now evidence that most of, if not all, the viral proteins are either modified by one or more of the Ubl systems or exert an effect on how these systems modify and regulate host proteins. While many details remain to be determined, it is clear that these protein modifier based post-translational systems are of central importance for HPVs to effectively co-opt the host cells for viral reproduction. Initial studies with high risk mucosal HPVs confirmed that targeted degradation of critical host cell growth regulatory proteins, such as p53 and pRB, was essential for the transformation process. Subsequent examinations of low risk mucosal types and cutaneous types have confirmed that utilization of the ubiquitin-proteasome system is a more general feature of HPVs to regulate levels of their viral proteins and to impact the host cell environment. Burgeoning studies on other Ubl systems, primarily the SUMO system, have revealed examples where HPVs modulate or utilize sumoylation to regulate viral activities. Additionally, there are novel examples of HPV proteins exhibiting cross-talk between the ubiquitin and sumoylation systems, which adds another level of complexity to the ability of HPVs to modulate the host cell growth and differentiation processes. Hopefully, the next 20 years will be as productive as the previous 20 years in fully unraveling the mechanisms by which HPVs utilize the ubiquitin family of modifiers to reprogram host cells.
